# Why peer-led crisis plan practice does — and does not — transform psychiatric ward culture: a realist-informed qualitative study from Japan

**DOI:** 10.3389/fpsyt.2026.1915695

**Published:** 2026-09-11

**Authors:** Mikie Ebihara, Michiko Ban, Tatsuya Tamura, Tomoko Omiya, Kumiko Ando

**Affiliations:** 1 The Jikei University School of Nursing, Chofu, Japan; 2 Jikei Nursing School, Minato-ku, Tokyo, Japan; 3 Faculty of Nursing, Fukushima Medical University, Fukushima, Japan; 4 Department of Public Health Nursing, Division of Health Science, Graduate School of Medicine, Tohoku University, Sendai, Japan; 5 Institute of Science Tokyo, Bunkyo, Japan

**Keywords:** coercive care, crisis plan, critical realism, epistemic injustice, Japan, morphogenetic approach, peer support worker, realist evaluation

## Abstract

**Background:**

Japan's psychiatric inpatient environment — characterized by the world's highest per-capita psychiatric bed rate, entrenched coercive care, and persistent physical restraint — has resisted policy reform. Crisis Plans (CP) are meant to be authored by the service users who may experience a crisis, but in practice, clinicians often write them instead. Peer-led CP practice has been proposed as a catalyst for recovery-oriented transformation, yet how it actually generates organizational change remains theoretically underspecified.

**Aim:**

This study aimed to identify the generative mechanisms and structural conditions through which peer-led CP practice transforms psychiatric ward culture in Japan.

**Methods:**

We interviewed thirteen nurses, managers, and peer support workers (PSWs) at two psychiatric hospitals. An integrated qualitative design was used: interview data were first analysed inductively using the Modified Grounded Theory Approach (M-GTA), then re-examined through Realist Evaluation (RE) to identify causal mechanisms. Archer's morphogenetic approach was applied to interpret how organizational structures change or persist.

**Results:**

Broad change in ward relationships developed when three mechanisms operated together: the hospital, rather than the individual nurse, taking responsibility for clinical risk (M1); the staged introduction of PSWs' lived-experience knowledge via a bridging staff member (M2); and staff letting go of the assumption that their job is to manage the person, allowing the peer-led return of CP authorship to the service user (M3). When these operated together, roles reversed: service users came to ask for care, and professionals responded. In the negative case (a site without PSWs), CP practice produced only individual-level change that was ultimately reabsorbed into existing practice.

**Conclusion:**

PSWs occupy a structurally distinctive position: standing between the ward's existing conditions and the processes that change them, they turn structural constraint into a driver of change. It appears to be the maintained tension and ongoing disagreement between professional and lived-experience perspectives that sustains this change and the self-questioning on which durable transformation depends.

## 1 Introduction

Over the past four decades, international mental health policy has shifted from institutional confinement toward community-based, recovery-oriented models (1, 2). Japan has been described as a structural outlier: it has been reported to hold the highest per-capita psychiatric bed count among OECD nations, with average inpatient stays exceeding 260 days and persistent concern over physical restraint rates (3).

This persistence appears historically constituted. From the 1950s, Japanese mental health policy expanded private psychiatric hospitals as a cost-effective substitute for public provision, institutionalising custodial care as a default response to mental illness (4–6). Subsequent deinstitutionalisation rhetoric has not been matched by the community infrastructure required to absorb discharged patients (3, 4). Beds built for custodial care have remained in use less because they were clinically necessary than because the system has not changed. Long-stay hospitalisation has persisted as the path of least resistance in a system that was never redesigned around recovery (3, 5).

A further structural feature is the individualised risk burden borne by frontline clinical staff. Japanese psychiatric nurses have been reported to express markedly lower safety perceptions than their European counterparts, associated with higher tolerance of physical restraint (7). This has been characterised not as a deficit of individual competence but as a structural condition in which clinical responsibility is constituted as personal, isolated, and indivisible (8, 9).

Crisis Plans (CP) — originating from Wellness Recovery Action Plan (WRAP) philosophy and grounded in Psychiatric Advance Directive (PAD) principles — were designed as rights-advocacy instruments authored by service users themselves (10). Peer-delivered WRAP has been associated in randomised trials with improved self-reported recovery and reduced symptoms (11). Joint Crisis Plans (JCPs) extend this framework through a co-produced document negotiated between service user and clinical team, with the explicit aim of reducing coercive intervention at the point of crisis (12). While CP implementation has been advancing internationally as a tool to address coercive care, implementation in Japan has been reported to diverge significantly: clinician-led risk management has been described as progressively taking over the CP format, reducing the plan to a symptom-management document that puts the avoidance of clinical risk ahead of the patient’s own wishes (13). This gap has been suggested to reinforce the risk-averse stagnation already embedded in the nursing environment.

Peer support workers (PSWs) have been internationally recognised as important resources for recovery-oriented practice (14, 15), yet their clinical role has been described as frequently marginalised — a situation where their knowledge is unfairly dismissed or devalued due to prejudices about their status as service users (a phenomenon Fricker (16) terms “testimonial injustice”). This vulnerability is argued to be especially acute in psychiatric settings (17).

Addressing this requires moving beyond individual attitude change to interrogate the organisational structures in which attitudes are embedded. Archer’s (18) morphogenetic approach provides a fitting theoretical frame. It treats organisational structures and the actions of the people within them as distinct forces that nevertheless shape one another, so that each can be examined in its own right (analytical dualism), and it traces their interaction across a four-phase sequence: T1, the structural conditions already in place before the intervention(structural conditioning);T2, the interactions among the people working under those conditions (social interaction);T3, the point at which those structures are either transformed (morphogenesis) or persist because the change has been absorbed rather than embedded (morphostasis); and T4, a further cycle in which an achieved transformation is itself reopened to question(critical re-morphogenesis). Archer’s (19, 20) account of meta-reflexivity — the capacity of people to question not only their conduct but the values on which it rests — informs the interpretation of T4 as a self-critical rather than merely reflective cycle.

Against this background, the present study asks why peer-led CP practice does — and does not — transform psychiatric ward culture. Specifically, it aims to identify the generative mechanisms and structural conditions through which peer-led CP practice may be associated with ward cultural transformation in Japanese psychiatric inpatient settings.

## 2 Methods

### 2.1 Research design

This study employed an integrated qualitative design combining M-GTA (21) with realist analytic logic drawn from RE (22), the latter grounded in a critical realist ontology (23). M-GTA yields actor-level description of meaning-making processes, while RE transforms those descriptions into causal explanations of why particular meanings arise under particular conditions. Their sequential integration — M-GTA first, RE second, each analytically complete before the next begins — represents the principal methodological feature of this study. Although grounded approaches and realist logic have more commonly been employed separately, their combination has an established precedent within the critical realist tradition, where grounded methods generate empirically anchored categories and a retroductive realist framework — reasoning back from observed patterns to the underlying mechanisms that could plausibly have produced them ―converts those categories into explanations that specify which combinations of context and mechanism produced which outcomes (24, 25). The sequential design adopted here preserves the inductive integrity of category generation while allowing realist re-specification to proceed without contaminating the initial coding. Archer’s morphogenetic approach (18) was applied as a *post-hoc* interpretive framework; Archer’s (20) concept of meta-reflexivity was applied to theorise T4 as a self-critical rather than merely reflective cycle.

Three analytically distinct actor layers were operationalised *a priori* to capture the multi-layered power structures and the unequal standing accorded to different kinds of knowledge anticipated within inpatient psychiatric settings. Layer I (frontline practitioners) was defined as clinical staff in direct contact with patients who individually bear the immediate burden of clinical risk at the frontline, structurally confined to managing crises within the ward’s normative coercive practices (Archer’s Primary Agents:shaped by the structure but not, individually, positioned to reorganise it). Layer II (organisational decision-makers) comprised staff holding formal authority over policy formulation and multidisciplinary coordination, possessing the institutional power to either maintain or transform the ward’s structural conditions (Corporate Agents: able to act collectively and deliberately on the structure). Layer III (peer support workers) represented peer staff whose lived experience of mental illness, combined with specialist training, grants them a structurally distinctive epistemic standpoint, enabling them to introduce experiential knowledge into clinical space to act as catalysts for cultural change (Social Actors: introducing a different kind of knowledge into the setting). The distinction matters analytically because it locates the capacity for structural change in Layer II, the burden of enacting care under existing constraints in Layer I, and the source of contesting knowledge in Layer III — an asymmetry that Section 3.3.3 shows to be productive rather than incidental.The specific professional roles recruited within each layer emerged from participant recruitment and are described in Section 2.2 (**Table 1**). Following the convention of RE, the elements of each explanation are labelled C (context), M (mechanism), and O (outcome), and a context–mechanism–outcome triad is abbreviated CMO. The specific contexts (C1–C3), mechanisms (M1–M3), and outcomes (O1a–O1c, O2) identified in this study are defined in Section 3.2 and summarised in **Table 2**.

**Table 1:** Participant characteristics, actor layer, site, and involvement in crisis plan (CP) and peer support worker (PSW) practice.

Part.	Speciality	Layer	Role	Site	Exp.	CP/PSW involvement	Min
A	Nurse	I	Head nurse, treatment-resistant/chronic ward	Y	20+yr	Oversight of all CP cases; PSW collaboration; zero-restraint initiative	50
B	Nurse	I	Senior nurse, treatment-resistant/chronic ward	Y	15+yr	2–3 CP co-support cases	40
C	Nurse	I	Head nurse, emergency/acute ward	Y	20+yr	Oversight; PSW study sessions; zero-restraint	30
D	Nurse	I	Staff nurse, emergency/acute ward	Y	5+yr	Frequent PSW contact; 1 direct CP collaboration	30
E	Nurse	I	Staff nurse, emergency/acute ward	Y	5+yr	1 direct CP support; PSW sessions	40
F	Nurse	I	Head, visiting nursing	Y	10+yr	7 CP co-support cases; post-discharge continuity	50
G	OT	II	OT dept head; PSW programme coordinator	Y	20+yr	Strategic design of PSW integration (Choco-WRAP)	40
H	MPSW	II	Head of medical social work	Y	20+yr	Community liaison; institutional PSW coordination	50
I	Psychiatrist	II	Hospital director	Y	30+yr	Formulated hospital-wide zero-restraint initiative	50
J	MPSW	II	Day-care unit chief	Z	10+yr	No PSW employed; CP/WRAP sole professional (negative case)	59
K	PSW	III	PSW lead	Y	10+yr	Lead PSW for CP/WRAP recovery programme	60
L	PSW	III	PSW staff	Y	~2yr	Implementing CP/WRAP (junior)	30
M	PSW (MPSW)	III	PSW staff; dual-role peer practitioner	Y	~5yr	CP/WRAP implementation; dual lived-experience and professional standpoint	40

PSW, peer support worker; MPSW, mental health and psychiatric social worker; CP, crisis plan; WRAP, Wellness Recovery Action Plan; Y, primary site; Z, negative-case site.

**Table 2:** M-GTA category structure and CMO classification.

No.	Category	Subcategory	Layer	CMO
1	Risk management that leaves individual staff to carry clinical risk alone	Nurses carrying clinical risk alone, and doubting that patients can recover	I-II	C1
		Checklist routines that overlook the individual patient	I-II	
2	A professional culture that trusts clinical knowledge more than lived experience, making it hard for PSWs to join the team	Professional knowledge trusted more than lived experience	I-II	C2
		PSWs kept outside the team, with staff and PSWs having no shared way of talking about care	I	
3	Service users losing control of their own crisis plans	Crisis plans taken over by clinicians and rewritten as risk-management documents	I-II	C3
		Clinician-written crisis plans experienced as a denial of the person's rights	II-III	
4	The organisation taking on clinical risk, and a move towards staying with patients instead of restraining them	The hospital, rather than the individual nurse, taking responsibility for clinical risk	I-II	M1
		Moving away from restraint and seclusion as the automatic response	I-II	
5	Working with PSWs changes which knowledge staff treat as reliable	Choco-WRAP sessions gradually making staff less certain of their own assumptions	II	M2
		PSWs' accounts leading staff to question what they had assumed	I-II	
		Staff choosing to stop treating their own knowledge as superior	I-II	
6	Staff come to see service users differently and rebuild how they work	Seeing service users as citizens who make their own decisions	I-II	M3
		Staff giving up the assumption that their job is to help someone who cannot manage alone	I-II	
7	Service users write their own plans, and ward relationships change as a result	Owning the crisis plan helps the person manage their own condition and feel safer	III	O1c
		Roles reversed: the service user asks for care, and professionals respond	II-III	
—	Ward-level change that appeared only when all three configurations operated together	Change that appeared only when all three configurations operated together, and not from any one alone	I-II-III	O2

**Figure 1:**
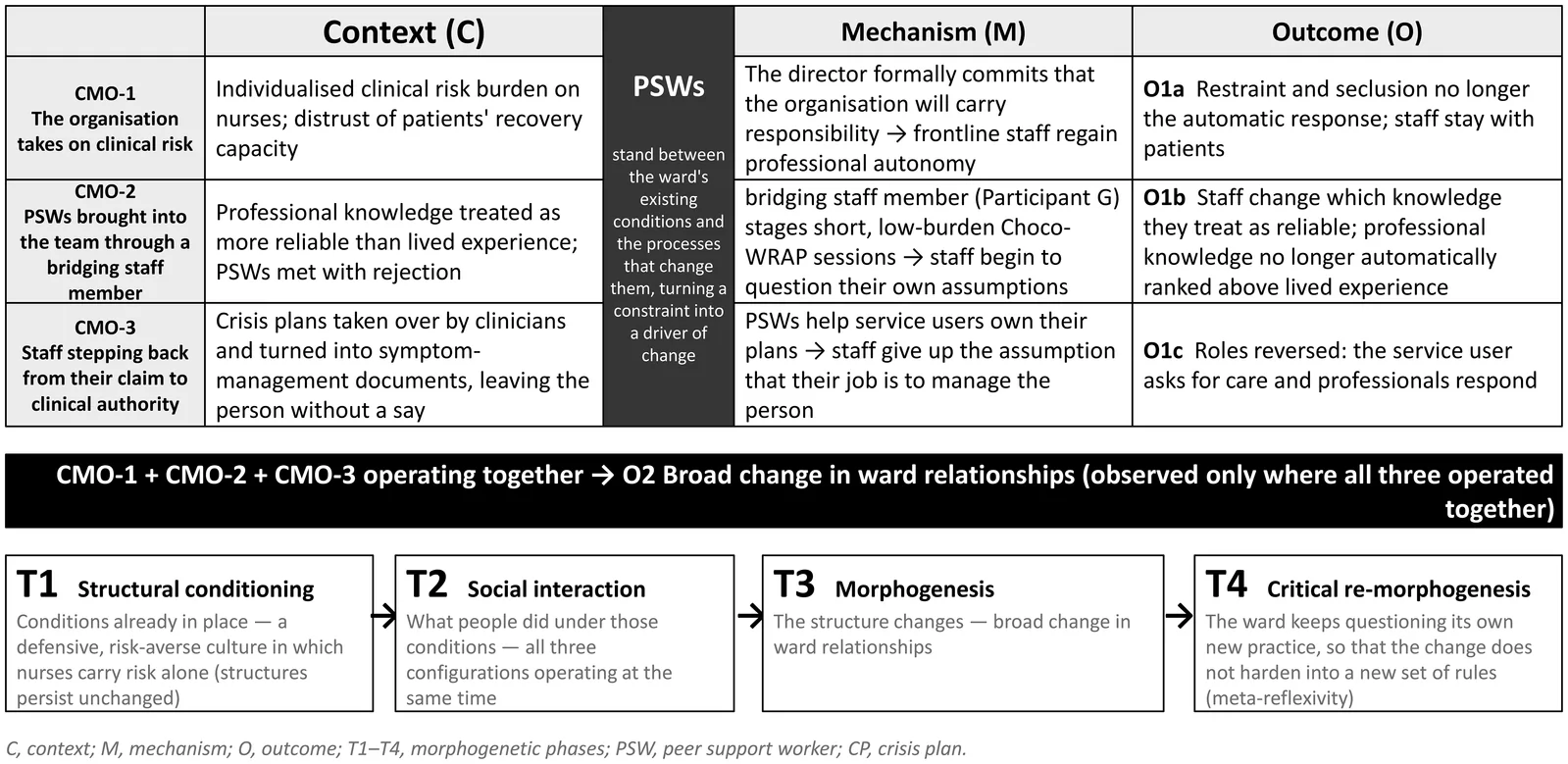
CMO configurations and the morphogenetic sequence (18–20). Because the negative case lacked all three mechanisms simultaneously, these data cannot isolate the contribution of any single mechanism. The findings suggest, more cautiously, that in the absence of full co-activation relationships may change without undergoing the reversal of roles observed at Site Y; this pattern should be read as suggestive rather than confirmatory.

CMO, context–mechanism–outcome; O1a–O1c, the proximate outcomes of CMO-1, CMO-2, and CMO-3 respectively (O1c being the reversal of positions between service user and professional); O2, the ward-level outcome that appeared only where all three CMOs operated together (an emergent property). The final row records this cross-cutting property (O2), which does not belong to any single category but arises across all three CMO configurations. Because participants described mechanisms and their immediate effects together, several categories contain material relating to both; the CMO column records the element to which each category was primarily assigned during classification, and **Figure 1** sets out the full configurations.

Key theoretical terms used throughout this paper are set out with plain-language definitions in **Box 1**.

Box 1 **Key theoretical terms and their plain-laguage meanings as used in this study.**

**Table:** 

Term	Plain-language meaning as used in this paper
Morphogenesis	The ward's structure actually changes.
Morphostasis	The ward's structure stays as it was, because the change was absorbed into existing practice.
Analytical dualism	Treating the ward's structures and the actions of the people in it as two separate things, so that each can be examined in its own right.
Meta-reflexivity	The capacity to question not only what one does, but the values behind it.
Emergent property	A feature of the whole that none of its parts has on its own.
Relational goods	Benefits that exist only within a relationship and cannot be credited to any one person.
Retroduction	Reasoning backwards from what was observed to the underlying processes that could have produced it.
Testimonial injustice	Someone's account being dismissed or given less weight because of prejudice about who they are.
Context-mechanism-outcome (CMO)	A way of stating: under these conditions, this process produced this result.
Primary Agents / Corporate Agents / Social Actors	People shaped by the structure but not able to reorganise it / people able to act together to change it / people who bring a different kind of knowledge into the setting.
Co-present care	Care in which staff stay with the patient rather than using restraint or seclusion.
Bridging staff member	A member of staff working where the professional and lived-experience worlds meet, who translates between them.
Choco-WRAP	A short, locally developed 15-minute version of WRAP that staff could join alongside their duties.
Shiensha-shu (支援者臭)	Literally "the odour of the helper": the wariness a person with lived experience feels towards the stance of the professional helper.

### 2.2 Participants

This study aimed to capture both the processes of ward cultural transformation and the structural factors shaping them from multiple perspectives. Because the pool of eligible facilities — psychiatric hospitals operating inpatient CP and WRAP programmes — is limited in Japan, convenience sampling was adopted. Eligible facilities were those that had introduced and were actively practising CP and WRAP programmes on inpatient wards.

Participants were recruited from two single-specialty psychiatric hospitals in Japan, selected through theoretical sampling along a single axis: the presence or absence of PSW involvement in CP/WRAP delivery. The primary site (Site Y), a single-specialty psychiatric hospital on the outskirts of a metropolitan area of more than two million residents, was selected as a hospital in which PSWs were involved in inpatient CP/WRAP delivery. Site Z, a single-specialty psychiatric hospital on the outskirts of a metropolitan area of more than three million residents, was not intended as a symmetrically matched comparison site but as a single theoretical negative case (26): a hospital practising CP/WRAP but without PSW clinical involvement, selected to examine what occurs when the candidate mechanisms (M1–M3) are absent under otherwise comparable contextual conditions. Within Site Z, CP/WRAP delivery rested with professional staff; the single informant (Participant J) occupied several vantage points relevant to the analysis, serving simultaneously as the professional operator of CP/WRAP, as a day-care unit chief with organisational oversight, and as a longitudinal observer of the service-user trajectories described in Section 3.3.2. Site Z was therefore treated as a bounded theoretical reference point rather than as the basis for symmetrical cross-site comparison. At Site Y, the hospital had an explicit organisational orientation toward reducing physical restraint and an established PSW-led CP/WRAP programme. At Site Z, although the hospital had begun employing individuals with lived experience of mental illness in non-clinical roles (e.g., cleaning and maintenance), PSWs had not been placed in programme delivery or direct patient care at the time of data collection.

Within each facility, the three actor layers defined in Section 2.1 were used as the sampling framework. Recruitment targeted participants whose differing positional knowledge, institutional authority, and experiential standpoints could illuminate the ward’s multi-layered dynamics from within each layer. Purposive sampling within layers continued until analytic sufficiency — the point at which further interviews within a layer yielded no new analytic content — was reached (27). The composition realised through this recruitment is summarised below and detailed in **Table 1**.

The recruitment yielded thirteen participants across the two sites: twelve at Site Y and one at Site Z. Layer I comprised six nurses, all at Site Y, ranging from staff nurses with around five years’ experience to head nurses with more than twenty. Layer II comprised four participants: at Site Y, an occupational therapist who headed the OT department and coordinated the PSW programme (Participant G), the head of medical social work (Participant H, MPSW), and the hospital director (Participant I, psychiatrist); at Site Z, the single informant, an MPSW serving as day-care unit chief (Participant J). Layer III comprised three peer support workers, all at Site Y, from a junior practitioner with roughly two years’ experience (Participant L) to the programme’s lead PSW (Participant K). Experience across the sample ranged from approximately two to more than thirty years. Participant G, the OT department head and PSW programme coordinator, is identified as the bridging staff member central to CMO-2 — a member of staff positioned at the interface between the professional and lived-experience domains, who mediates between the two (28); Participant J, at Site Z, served as the sole Layer II informant and is the source of the negative-case data; and Participant M holds a dual position as both a person with lived experience of psychiatric illness and a trained MPSW, occupying a structurally distinctive standpoint within Layer III. Full participant characteristics are presented in **Table 1**.

### 2.3 Data collection

Semi-structured interviews were planned as the primary data collection method, audio-recorded with informed consent. Interview duration was not fixed in advance but guided by the depth of participants’ accounts. Interview guides were developed separately for each actor layer and covered: experiences of CP and PSW collaboration; perceived changes in ward culture and professional practice; structural conditions enabling or constraining transformation; and the meanings attributed to crises and recovery across professional boundaries. Reporting followed COREQ (29) for the qualitative interview components and the RAMESES II reporting standards (30) for the realist components. Because realist logic was applied in a focused second analytic phase rather than through iterative refinement of a programme theory, this study is best understood as a realist-informed analysis rather than a full realist evaluation; RAMESES II accordingly guided the transparent specification and reporting of the context–mechanism–outcome configurations rather than the conduct of a complete evaluation cycle. Interviews were conducted in October and November 2025, with a mean duration of 44 minutes (range: 30–60 minutes).

### 2.4 Analytic process

Analysis proceeded in three sequential phases, each analytically complete before the next began.

Phase 1 (M-GTA). Concepts were generated inductively from interview transcripts using M-GTA structured worksheets (21). Each worksheet recorded: (a) the concept name and operational definition; (b) empirical variations across participants’ narratives; (c) contrasting or disconfirming instances; and (d) analytic memos documenting interpretive reasoning. Analytic sufficiency (27) was used as the completion criterion.

Phase 2 (RE). Only after M-GTA analytic sufficiency was confirmed did the analytic team transition to RE. The seven M-GTA categories were classified into C, M, or O by applying three sequential questions: (C) Does this category describe conditions structurally present before the PSW-led intervention? (M) Does this category describe the process that generated change? (O) Does this category describe results produced by mechanism activation? Classification was conducted independently by two researchers; disagreements were resolved by returning to primary data.

Phase 3 (Abduction). The identified mechanisms were matched against existing theoretical literature through abductive reasoning. Archer’s (18) morphogenetic framework was applied to position each CMO within a temporal sequence (T1 → T2 → T3 → T4).

### 2.5 Rigour

The multidisciplinary research team comprised: ME (lead analyst), responsible for overall research design, primary analysis, and synthesis; KA, a researcher with expertise in Japanese psychiatric care systems and hospital organisational culture, serving as contextual auditor; MB, a clinical specialist in CP facilitation and PSW practice; and TO and TT, nursing researchers specialising in PSW practice and peer support in mental health, who provided methodological scrutiny and independently cross-checked the RE classification.

## 3 Findings

### 3.1 Overview

Participant and setting characteristics are described in the Methods (Section 2.2 and **Table 1**). The following sections present the category structure and CMO classification (3.2), the three CMO configurations (3.3.1), the negative-case analysis (3.3.2), and the cross-cutting dynamic (3.3.3).

### 3.2 Category structure and CMO classification

M-GTA analysis generated 24 concepts converging into seven categories across 15 subcategories. CMO classification was applied *post-hoc* (**Table 2**). The following typographic conventions are used throughout Section 3. Category names appear in bold capitals where they head a subsection, and in sentence case in **Table 2** and **Figure 1**, where the full labels would otherwise be difficult to read. Subcategory names appear in quotation marks, and direct participant quotations in italics within quotation marks, followed by the participant identifier.

CATEGORY 1: RISK MANAGEMENT THAT LEAVES INDIVIDUAL STAFF TO CARRY CLINICAL RISK ALONE (C1)

This category describes the risk-averse, control-oriented culture established in the ward. It comprises two subcategories: “Nurses carrying clinical risk alone, and doubting that patients can recover” and “Checklist routines that overlook the individual patient.” Frontline nurses were structurally positioned as the sole bearer of risk when patients became agitated or expressed self-harm, suicidal, or aggressive impulses — responsible for anticipating, containing, and answering personally for any adverse outcome, up to and including a patient’s death, without organisational recourse. This individualised risk structure left coercive management as the default: there was no institutional permission to do otherwise.

“The standard excuse for not acting is: ‘Who’s going to take responsibility for that?’” — Participant A

“Even when the probability is low, risk is always there in some form.” — Participant B

Yet when nurses began attending Choco-WRAP sessions and hearing PSWs describe their own experiences of agitation and de-escalation, something shifted. Choco-WRAP — a locally developed, abbreviated form of WRAP designed so that staff could join brief 15-minute sessions alongside their duties rather than as a separate patient-only programme — served as the low-burden entry point for this encounter. Patients managed through restraint or isolation were reconceived as people whose distress could be met relationally. The ward’s physical environment began to change: a dedicated unit area was established where patients could move more freely, a vending machine was installed to allow informal moments of relaxation, and the assumption that these changes would generate incidents proved unfounded.

“Since we set up the unit area, episodes of agitation have decreased significantly.” — Participants C and E

“Patients who previously needed seclusion stabilised more readily.” — Participant D

“At first, everyone insisted smartphones were ‘dangerous.’ Now we let patients keep their charging cables on the ward.” — Participant C

CATEGORY 2: A PROFESSIONAL CULTURE THAT TRUSTS CLINICAL KNOWLEDGE MORE THAN LIVED EXPERIENCE, MAKING IT HARD FOR PSWs TO JOIN THE TEAM (C2)

It comprises two subcategories: “Professional knowledge trusted more than lived experience” and “PSWs kept outside the team, with staff and PSWs having no shared way of talking about care. “ For some staff, resistance to PSWs joining the team was not only about procedures. Nurses with long careers in the hospital reacted as though a line was being crossed: if a former patient could work as a colleague, the boundary that had defined them as professionals no longer held.

“Among the more senior nurses, there was strong resistance to PSWs joining the team. Some said: ‘I can’t work alongside someone who used to be a patient. If that’s how it’s going to be, I’ll quit.’” — Participants C and G

“The past gets in the way. There’s a lack of imagination.” — Participant A

“When we try something new, the response is: ‘Why should we have to do that when we’re already this busy?’” — Participant A

“We moved to hire and place PSWs on the ward without laying the groundwork first. The result was that neither professionals nor peers could cope with each other’s presence, and both sides were exhausted.” — Participant H

CATEGORY 3: SERVICE USERS LOSING CONTROL OF THEIR OWN CRISIS PLANS (C3)

This category describes how CP practice moved away from its original purpose as a rights instrument. It comprises two subcategories: “Crisis plans taken over by clinicians and rewritten as risk-management documents” and “Clinician-written crisis plans experienced as a denial of the person’s rights”.

“A crisis plan authored by clinicians is like signing someone else’s donor card for them. If I decide to donate my organs to someone who needs it, that makes sense. But if the clinician decides and asks me to sign, that is a different thing entirely. Mental health is the same question: whose life is this?” — Participant K

Participant M, drawing on interviews with dozens of service users and on their own CP authorship experience, named the structural logic of this distortion from the lived-experience standpoint:

“When I listened to colleagues talk about CP, I kept hearing: we need a plan to stop things getting worse. From the perspective of someone with lived experience, that carries a very strong smell of the helper — shiensha-shū. What we want from CP is self-advocacy and self-determination first.” — Participant M

The term shiensha-shū — literally the “odour of the helper” — warrants explanation for readers outside this context. It is not a description of a stated attitude but of something sensed: the almost physical wariness that a person with lived experience of psychiatric care comes to feel toward the posture of the professional helper. Significant here is that the term was voiced by a PSW who is simultaneously a service user. The metaphor of smell is used deliberately. The term does not describe a conclusion the person reaches by reasoning; it describes a distrust that is noticed immediately, in the way a smell is noticed. It is a wariness built up over years of being treated as someone to be helped, however well meant that help was. The diagnostic power of the concept lies precisely there: a clinician-authored CP can be procedurally faultless and still carry this odour, because what the service user detects is not the document’s content but the relational stance from which it was written. Naming shiensha-shū thus gives the lived-experience standpoint an instrument for detecting, beneath the surface of participatory language, the re-centring of the helper’s logic.

CATEGORY 4: THE ORGANISATION TAKING ON CLINICAL RISK, AND A MOVE TOWARDS STAYING WITH PATIENTS INSTEAD OF RESTRAINING THEM (M1)

This category describes how nurses were released from carrying clinical risk on their own. It comprises two subcategories: “The hospital, rather than the individual nurse, taking responsibility for clinical risk” and “Moving away from restraint and seclusion as the automatic response.” Co-present care here means care in which staff stay with the patient rather than using restraint or seclusion.

“The moment we removed the restraint straps from the ward and started from the assumption that ‘these things simply don’t exist here,’ our entire response to patients’ crises changed dramatically.” — Participant A

“I am not acting because management told me to. I act on my own conviction that this CP is indispensable to the patient’s recovery.” — Participant B

In practical terms, this redistribution was enacted through concrete organisational arrangements rather than exhortation. The hospital director’s hospital-wide zero-restraint initiative (Participant I) established, as formal institutional policy, that responsibility for adverse outcomes would be borne by the organisation rather than devolved onto the individual nurse at the bedside. This was materialised in the removal of physical-restraint equipment from the ward and in an explicit organisational premise that coercive intervention was no longer the default recourse — arrangements that together relieved frontline staff of the isolated accountability described in C1. Because the guarantee was located in managerial authority and standing policy rather than in individual discretion, nurses could reorient from defensive compliance toward recovery-oriented action undertaken on their own conviction, as the accounts below reflect.

CATEGORY 5: WORKING WITH PSWs CHANGES WHICH KNOWLEDGE STAFF TREAT AS RELIABLE (M2)

This category describes how professionals’ assumptions about whose knowledge is reliable were unsettled, and how they came to trust that service users can recover. It comprises three subcategories: “Choco-WRAP sessions gradually making staff less certain of their own assumptions,” “PSWs’ accounts leading staff to question what they had assumed,” and “Staff choosing to stop treating their own knowledge as superior”.

“I was meticulous about not generating resistance or rejection among the professionals. That’s precisely why I strategically proposed the ‘15-minute’ format [Choco-WRAP], reducing the psychological and time burden to an absolute minimum.” — Participant G

“I started attending out of a sense of organisational obligation, but before I knew it I was looking forward to what story I would hear next.” — Participant D

The transformation often began at the bedside, in moments of open confrontation. One nurse recalled a patient rounding on her in anger:

“A patient once turned on me, almost shouting — ‘How could someone like you possibly understand what I feel!’ And in that moment I realised, honestly, that I could not. But the fact that there is someone on this ward — a PSW — who can truly understand that pain is itself decisively important for care.” — Participant D

Crucially, this resonance was not merely attitudinal. Because nurses were the staff in most sustained daily contact with patients, they were also the ones who most directly bore the patient’s distress when it erupted as anger, fear, or refusal. By standing between the patient and the clinical team, the PSW translated that distress into terms the nursing staff could act upon. Where a nurse had previously faced a patient’s agitation alone and without a script, the PSW’s mediation made the patient’s wants legible, allowing the nurse to know what concrete response the situation called for. The epistemic shift thus carried a practical corollary: nurses did not only come to see patients differently, but came to know what to do.

“Having a PSW stand in between, helping the patient say the hard-to-say things, increased the patient’s conviction and made it easier for them to consult with us.” — Participant G

“The PSW stops me right when I’m about to become overbearing.” — Participant D

CATEGORY 6: STAFF COME TO SEE SERVICE USERS DIFFERENTLY AND REBUILD HOW THEY WORK (M3)

This category describes how professionals’ sense of their own role changed, so that service users were no longer seen as people to be managed but as citizens who make their own decisions. It comprises two subcategories: “Seeing service users as citizens who make their own decisions” and “Staff giving up the assumption that their job is to help someone who cannot manage alone”.

“The team transformed into an autonomous collective that questions existing routines and continues to ask ‘Is this really right?’ — never stopping the inquiry.” — Participant A

CATEGORY 7: SERVICE USERS WRITE THEIR OWN PLANS, AND WARD RELATIONSHIPS CHANGE AS A RESULT (O1c)

This category describes the change in ward relationships that followed. It comprises two subcategories: “Owning the crisis plan helps the person manage their own condition and feel safer” and “Roles reversed: the service user asks for care, and professionals respond” (O1c).

“Before I gave the CP to my psychiatrist, when I asked for the medication and was stopped, it felt like something was being done to me from outside. After I gave the CP, it became: my psychiatrist was responding to what I had asked for. If you filmed it with a silent camera, you would see the same scene. But the subject had changed — the origin had changed — and that is a completely different world.” — Participant K

“Recovery means becoming the author of your own story. Before CP, the authors were the clinicians and the administrators — my experiences were translated into diagnostic categories and returned to me. CP gave me back the pen.” — Participant K

“After submitting a crisis plan to the ward, it is the patient who is now ‘the one making requests’ in care, while the psychiatrist and other professionals are ‘the ones responding to those requests.’ Subject and object have completely exchanged positions.” — Participant J

### 3.3 CMO configurations

#### 3.3.1 Three CMO configurations

[CMO-1] The organisation takes on clinical risk: In a defensive ward culture in which clinical risk fell on individual practitioners (C1), the hospital director’s formal commitment that the organisation would carry responsibility (M1) released frontline staff from carrying that risk alone. This made it possible to stop using restraint and seclusion as the automatic response and to move towards staying with patients (O1a).

[CMO-2] PSWs brought into the team through a bridging staff member In a setting where professional knowledge was trusted more than lived experience and PSWs faced barriers to joining the team (C2), the bridging staff member (Participant G) introduced short, low-burden Choco-WRAP sessions (M2), which changed which knowledge staff treated as reliable (O1b).

[CMO-3] Staff stepping back from their claim to clinical authority: In a setting where crisis plans had been rewritten by clinicians in ways that left service users without a say (C3), as PSWs supported service users in genuinely owning their own plans, staff gave up the claim that judging what the person needed was theirs to make (M3). Service users moved from being managed to asking for the care they wanted, so that who initiates and who responds in the care relationship was reversed (O1c).

The findings suggest that these three CMO configurations may not operate independently. Broad change in ward relationships (O2) appeared only where all three were co-activated. Given the study design, however, it cannot be established whether O2 required all three configurations, reflected an additive combination of the mechanisms, or was influenced by unmeasured differences between the two sites.

#### 3.3.2 Negative-case analysis (site Z)

At Site Z, CP/WRAP was delivered by professional staff without PSW involvement, and the analysis draws on Participant J, who occupied several relevant vantage points (operator, organisational decision-maker, and longitudinal observer). The contextual conditions C1 and C3 appeared structurally present in a form comparable to Site Y, and Participant J demonstrated acute conceptual awareness of both. Yet the candidate mechanisms M1–M3 were absent, and the three outcomes O1a–O1c were not observed to activate at the structural level. Site Z is presented here as a single theoretical negative case rather than as a symmetrical comparator.

Two service-user trajectories show what CP achieved under these conditions, and what it failed to protect. Service User P, a long-standing service user living with a physical disability, engaged CP with directness: he articulated his own crisis signals, arranged his own gym membership, and spoke openly about his life. He was subsequently transferred to a residential care facility on grounds of physical disability. Whether the possessions, routines, and freedoms he had built around his CP were permitted in the new setting, Participant J did not know.

“I wonder how much of that kind of thing — the leisure, the enjoyment — was actually allowed there.” — Participant J

According to Participant J, the CP Service User P had authored carried no formal standing in the receiving facility. These details derive from a single retrospective account and could not be independently verified; they are presented here as illustrative rather than evidential. The trajectory nonetheless raises the possibility that a rights instrument that is not protected by any structure may end up as no more than a record of what the person once wanted, kept in a file that may not follow them when they move.

Service User Q recorded employment, marriage, and licence recovery as CP goals. The facility head brought him into a peer exercise group and gave him a role in which the other members took their lead from him. At the time of interview, Q was in work and had sustained a relationship for about a year — two of the three goals he had written into his CP quietly and partially realised.

“For now, at least, he seems to be managing in roughly the form he himself had hoped for; all I can do is watch over it.” — Participant J

These changes did not originate in Q alone but emerged within a web of relationships linking Participant J, the facility, Q himself, and the wider day-care community, and can be read as the nascent form of what Donati and Archer (31) term relational goods — properties of a relationship that cannot be attributed to any single party. Yet Site Z had no structures that could take up these gains, extend them, and build them into how the service worked. Without M1–M3 the gains had nothing to hold them in place: the conditions alone, without these processes, could not sustain change that had occurred between individuals.

“However we go about it, unless the person opens themselves to us there is nothing we can do. I don’t think either of them showed us everything — only the parts they judged it was alright to show.” — Participant J

This is reflexive agency at the interpersonal level: Participant J recognised that what he could know — and therefore hold — was bounded by what each service user chose to disclose, and located the gains in the service users themselves rather than in his own provision. Because Site Z had no peer worker whose lived experience could be set against the professional viewpoint, Participant J had no alternative viewpoint against which to test his own thinking.

He could doubt his own knowledge, but no standpoint existed from which the helper’s role itself was routinely called into question. His awareness therefore remained a personal quality rather than a self-critical process built into how the ward operated — what Archer terms meta-reflexivity (T4). The negative case thus shows not an absence of reflection but its limit: crisis-plan practice can prompt reflection between individuals even without mechanisms M1–M3, but without the structural conditions that sustain this collective self-questioning, such change is not embedded in the ward and is liable to be reabsorbed into existing practice.

#### 3.3.3 Cross-cutting dynamic

A structural asymmetry runs across all three CMO configurations: Layer II holds institutional authority but lacks lived-experience perspective; Layer I holds frontline practical knowledge but lacks organisational power; Layer III carries experiential knowledge irreducibly grounded in having personally navigated psychiatric crisis, but lacks institutional protection. In Archer’s terms, the capacity to reorganise the structure (Corporate Agents), the burden of acting within it (Primary Agents), and the knowledge capable of contesting it (Social Actors) are held by three different sets of people, and no single layer can produce transformation alone.The transformative potential resides not in resolving these asymmetries but in their productive, maintained tension.

Participant G’s bridging staff member function (28) appeared to be the mechanism through which this tension became generative. T4 — the cycle in which the ward keeps questioning its own new practice, so that the change does not harden into a new set of fixed rules was instantiated through Participant M’s sustained critique of shiensha-shū and Participant K’s extension of that critique to PSW practice itself:

“A PSW is still a specialist — not the person. If the PSW becomes the author of the CP, we have simply replaced one professional narrator with another. What we can do is describe what the tool is, share how it functioned in our own experience, and ask: how might you use it? The rest belongs to the person.” — Participant K

This dynamic corresponds to Archer’s (20) meta-reflexivity and is discussed further in Section 4.

## 4 Discussion

### 4.1 A morphogenetic account of psychiatric ward cultural transformation

This study’s findings are consistent with the view that psychiatric ward cultural transformation may follow a morphogenetic sequence (18) rather than a co-constitutive or linear process. The three CMOs each appeared to contain Archer’s temporal chain — T1 → T2 → T3 — and their operating together was associated with O2, a ward-level outcome that none of the three produced on its own (an emergent property: a feature of the whole that none of its parts has by itself).

The single theoretical negative case at Site Z provides an important qualification rather than a symmetrical comparison. CP appeared to generate relational emergence at the interpersonal level, but without M1–M3 this emergence was not observed to propagate to structural transformation. As these two trajectories derive entirely from a single informant’s retrospective, second-hand account and could not be independently verified, the following interpretation is offered as illustrative rather than evidential. This absorption may not simply mean that good outcomes failed to appear: in at least one case it appeared to have real consequences for the person’s life. Service User P built aspects of a life through CP, the gym, and public self-expression — only to be transferred, on grounds of physical disability, to a setting that had no stated obligation to maintain any of it. They raise the possibility that relational goods may dissolve at the boundary of the relationship that generated them. This pattern may illustrate morphostatic absorption — not a dramatic reversal of change, but its gradual elimination through routine administrative procedures.

We tentatively term the joint operation of all three CMOs ‘CMO co-activation’ and suggest this may represent a candidate extension of RE theory concerning the conditions under which complex social interventions may produce morphogenetic rather than morphostatic outcomes (18, 22). The identification of T4 represents a further theoretical proposition, tentatively theorised as an organisational instantiation of Archer’s (20) meta-reflexivity.

It is at this point that the analytic value of Archer’s framework becomes most apparent, and that the concept of shiensha-shū acquires its theoretical weight. Archer’s (20) meta-reflexivity is, in the abstract, the capacity of agents to subject their own value commitments to critical scrutiny rather than merely to monitor their conduct. The present data suggest that shiensha-shū may be read as the clinical embodiment of precisely this capacity. When a person with lived experience senses the “odour of the helper”, they are picking up an early sign that the ward is drifting back to its previous way of working: the point at which the professional’s way of thinking starts to move back to the centre and the new practice begins to harden into a fixed routine.

Crucially, the data do not suggest that this odour was eliminated once nurses became more receptive and PSWs were accepted onto the ward. Rather, it appeared to persist beneath the observable changes. That it persisted is not a sign that the change was incomplete; it served a purpose.

If the difference between the professional and the lived-experience viewpoint disappeared, there would be nothing left for staff to test their assumptions against, and the ward would be likely to return to its earlier way of working — this time with a friendlier appearance.

The maintained tension that Participant M and Participant K sustained — turning the critique of shiensha-shū back even upon PSW practice itself — is therefore not a sign of incomplete transformation but the mechanism by which transformation is kept open. On this reading, shiensha-shū works as an ongoing check that keeps the change open to revision. It is a sensitivity grounded in lived experience that keeps noticing — and therefore keeps challenging — the repeated tendency for the professional’s way of thinking to move back to the center. The pairing of an integrated qualitative method with Archer’s morphogenetic approach thus did analytic work that neither could have done alone: the method surfaced shiensha-shū as a grounded concept, while the morphogenetic frame elevated it from an individual sensibility into a reflexive mechanism through which the service user’s right to authorship may be defended over time.

### 4.2 Peer-led CP as a structurally novel intervention

The present findings suggest that peer-led CP may be structurally distinct from some existing peer support models. In complementary models, PSW knowledge may enter clinical space as an addendum to professional knowledge, leaving the epistemic hierarchy largely intact. In the model observed here, PSW knowledge appeared to be constitutive of the clinical process itself, with the potential to contest that hierarchy. “Peer-led” here denotes the centrality of lived-experience knowledge to the CP process rather than peers initiating or driving the intervention: its introduction was professionally brokered through a bridging staff member (CMO-2), and the term should not be read as “peer-driven.” This is consistent with the stance, voiced by Participants M and K, that the peer’s role is to describe the tool and hand authorship to the service user rather than to become its new author.

Participant K articulated the core principle of this intervention: what distinguishes CP from clinician-authored treatment plans is not the document’s content but its authorship. A clinician-authored CP substitutes the clinical narrative for the person’s own while presenting that substitution as participation. Participant M’s concept of shiensha-shū names the same condition from within the facilitation process: the moment the helper’s logic re-centres the clinical framework, the CP ceases to be a rights instrument and becomes its opposite.

Participant K further introduced the distinction between crisis and emergency, and between crisis management and risk management. Crisis, in the developmental sense following Caplan (32), may be understood not as a condition to be prevented but as one to be navigated: a plan primarily oriented toward eliminating crisis may also close off the growth that navigating a crisis can produce. This perspective, if substantiated, would suggest that clinician-authored CP does not merely fail to produce O1c but may actively prevent it — though this interpretation rests on limited data and warrants further investigation.

### 4.3 Limitations

Several limitations warrant consideration. First, the historical context of Japanese psychiatric institutionalisation is reconstructed from existing literature rather than original data. Second, the study is subject to single-site and best-case bias, since both sites already operated CP/WRAP programmes and Site Y in particular represented a setting with unusually strong organisational commitment. Third, the temporal sequence proposed is reconstructed retrospectively from participant accounts rather than observed prospectively. Fourth, the bridging staff member function described in CMO-2 was embodied in a single individual, and its transferability across settings remains unclear. Fifth, and most consequentially, this study was not a symmetrical two-site comparison. Site Z functioned as a single theoretical negative case, and its data derive from one Layer II informant; although that informant occupied several relevant vantage points, no Layer I or Layer III data were collected at Site Z. The findings concerning Site Z should therefore be read as a bounded illustration of what may occur in the absence of M1–M3, not as a generalisable account of PSW-absent settings, and the positive claims of this study are grounded in the Site Y data. Finally, the trajectories of Service Users P and Q are reconstructed entirely from Participant J’s retrospective account; no direct service-user data were collected, and these trajectories should be interpreted with particular caution.

### 4.4 Transferability

The transferability of these findings warrants careful framing. The structural conditions identified here — extreme bed dependency, individualised risk burden, and a marginalised PSW role — are particularly pronounced in the Japanese context, which might suggest limited applicability elsewhere. We suggest, however, that it is the specific combination of contexts and mechanisms rather than the specific contextual values that may travel: the proposition that peer-led CP requires simultaneous organisational risk redistribution, bridging staff member mediation, and genuine CP ownership to produce relational transformation is, in principle, testable in any setting where coercive defaults and epistemic hierarchies obtain. The Japanese case may thus function less as an exception than as an amplified instance in which these conditions become unusually visible.

A further caveat concerns the scope of the intervention itself. This study observed ward cultural transformation solely through the lens of peer-led CP and WRAP practice; it cannot speak to whether other recovery-oriented interventions might activate comparable mechanisms. Given the diversity of recovery-oriented programmes now in use, the combined theoretical framework proposed here is best read as a hypothesis grounded in one intervention modality — plausibly, but not demonstrably, relevant to others — and its applicability beyond CP/WRAP remains an open empirical question.

## 5 Conclusion

Returning to the question posed at the outset — why peer-led CP practice does, and does not, transform psychiatric ward culture — this study suggests that the answer lies less in the presence of programme elements than in whether three structural mechanisms are co-activated.

This study offers a preliminary morphogenetic account of psychiatric ward cultural transformation. Three candidate CMO configurations were identified: CMO-1 (the organisation takes on clinical risk → frontline staff regain professional autonomy); CMO-2 (lived-experience knowledge introduced in stages through a bridging staff member → a change in which knowledge staff treat as reliable); CMO-3 (staff give up the assumption that their job is to manage the person, as PSWs support service users in owning their plans → roles reversed between service user and professional). The principal finding is that broad change in ward relationships (O2) appeared only where all three configurations operated together, and not through any one of them alone.

These findings tentatively suggest that the social implementation of peer-led CP may depend on several structural conditions rather than on programme elements alone: (1) formal management commitment to organisational risk redistribution (CMO-1); (2) a designated bridging staff member with institutional protection (CMO-2); (3) a critical mass of PSWs with CP experience (CMO-3); and (4) institutionalised critical tension to limit the tendency for transformation to solidify into new orthodoxy (T4).

The present findings suggest that PSWs may hold an unusual position in the ward: they stand between the conditions that already exist and the processes that change those conditions, and can turn a constraint into something that drives change. This is not easily achieved through staff training or policy instruction alone. In Archer’s terms, it is the maintained tension between professional and lived-experience standpoints — not its resolution — that appears to generate the emergence and reflexivity on which lasting change depends; PSW-led CP may be understood as one clinical means by which that tension is built into practice and kept productive.

This study does not claim that peer-led CP universally or inevitably produces the outcomes described, nor that the role of PSWs cannot be supported or developed through training and organisational investment. Rather, the findings suggest that the relational goods associated with PSW-led CP practice in this study — in Donati and Archer’s (31) sense — appeared to be produced within lived relationships, and that replication may require attending to the structural conditions identified here rather than to the programme elements alone. These propositions remain tentative and invite scrutiny through further research across broader settings.

## Data Availability

The datasets generated and analysed for this study are not publicly available because they consist of qualitative interview transcripts containing potentially identifying information, and the consent obtained from participants and the approval granted by the research ethics committee do not permit unrestricted data sharing. Anonymised excerpts may be made available by the corresponding author on reasonable request, subject to approval by the research ethics committee of The Jikei University School of Medicine.
